# Supplementary material for: Machine Learning Accelerates Crystallization for Structure Determination
Source: Angew Chem Int Ed Engl. 2026 May 4;65(25):e1218503. doi: 10.1002/anie.1218503 (PMC13266958; doi:10.1002/anie.1218503)

checkCIF/PLATON report

Structure factors have been supplied for datablock(s) lcz088

THIS REPORT IS FOR GUIDANCE ONLY. IF USED AS PART OF A REVIEW PROCEDURE FOR PUBLICATION, IT SHOULD NOT REPLACE THE EXPERTISE OF AN EXPERIENCED CRYSTALLOGRAPHIC REFEREE.

No syntax errors found. CIF dictionary Interpreting this report

Datablock: lcz088

Bond precision:	C-C = 0.0217 A		Wavelength=1.54184
Cell:	a=13.0986(2)	b=22.5585(3)	c=25.0454(4)
	alpha=109.326(1)	beta=92.498(1)	gamma=90.352(1)
Temperature:	100 K		

	Calculated	Reported
Volume	6975.23(18)	6975.22(18)
Space group	P -1	P -1
Hall group	-P 1	-P 1
Moiety formula	C21 H7.39 Ag2.83 Cl0.57 F17 N6.24 S0.57, 3.055(C15 H3 Ag3 F18 N	3.437(C17.215 H3.443 Ag3.443 F20.658 N6.886), 3.337(C12 H8 Cl N
Sum formula	C108 H44 Ag12 Cl4 F72 N28 S4	C108 H44 Ag12 Cl4 F72 N28 S4
Mr	4666.19	4666.19
Dx,g cm-3	2.222	2.222
Z	2	2
Mu (mm-1)	15.876	15.876
F000	4464.0	4464.0
F000’	4489.93	
h,k,lmax	16,28,31	16,28,31
Nref	30058	28369
Tmin,Tmax	0.034,0.079	0.442,1.000
Tmin’	0.006	

Correction method= # Reported T Limits: Tmin=0.442 Tmax=1.000
AbsCorr = MULTI-SCAN

Data completeness= 0.944 Theta(max)= 78.603

R(reflections)= 0.1102(20741) wR2(reflections)= 0.2671(28369)

S = 1.048 Npar= 3060

The following ALERTS were generated. Each ALERT has the format
test-name_ALERT_alert-type_alert-level.
Click on the hyperlinks for more details of the test.

Alert level B

```
PLAT342_ALERT_3_B Low Bond Precision on  C-C Bonds ..............    0.02165 Ang.
```

Author Response: Disordered structure.

```
PLAT971_ALERT_2_B Check Calcd Resid. Dens.  0.93Ang From Ag2            2.61 eA-3
```

Author Response: This Alert is due to presence of residual density in the presence of heavy metal atom (Ag).

Alert level C

```
PLAT042_ALERT_1_C Calc. and Reported MoietyFormula Strings  Differ     Please Check
              Calc: C21 H7.39 Ag2.83 Cl0.57 F17 N6.24 S0.57, 3.055
                    (C15 H3 Ag3 F18 N6), 3.431(C12 H8 Cl N S)
              Rep.: 3.437(C17.215 H3.443 Ag3.443 F20.658 N6.886),
                    3.337(C12 H8 Cl N S), C8.79 H5.47
PLAT082_ALERT_2_C High R1 Value .................................       0.11 Report
PLAT084_ALERT_3_C High wR2 Value (i.e. > 0.25) ..................       0.27 Report
PLAT088_ALERT_3_C Poor Data / Parameter Ratio ....................       9.27 Note
PLAT250_ALERT_2_C Large U3/U1 Ratio for <U(i,j)> Tensor(Resd    2)        2.8 Note
PLAT250_ALERT_2_C Large U3/U1 Ratio for <U(i,j)> Tensor(Resd    3)        2.7 Note
PLAT362_ALERT_2_C Short  C(sp3)-C(sp2) Bond  C10      - C46      .       1.41 Ang.
PLAT362_ALERT_2_C Short  C(sp3)-C(sp2) Bond  C14      - C22      .       1.41 Ang.
PLAT721_ALERT_1_C Bond    Calc     1.37(7), Rep     1.39000 Dev...       0.02 Ang.
              C121     -C126           1_555   1_555 ........     #  331 Check
PLAT721_ALERT_1_C Bond    Calc     1.37(7), Rep     1.39000 Dev...       0.02 Ang.
              C123     -C124           1_555   1_555 ........     #  333 Check
PLAT723_ALERT_1_C Torsion Calc      -2(10), Rep        0.00 Dev...       2.00 Sigma
              C19 -C20 -C21 -C16    1_555  1_555  1_555  1_555      #  282 Check
PLAT906_ALERT_3_C Large K Value in the Analysis of Variance ......     28.898 Check
PLAT906_ALERT_3_C Large K Value in the Analysis of Variance ......      5.445 Check
PLAT906_ALERT_3_C Large K Value in the Analysis of Variance ......      2.680 Check
PLAT911_ALERT_3_C Missing FCF Refl Between Thmin & STh/L=    0.600        278 Report
               -1  1  0,   1  1  0,   0  2  0,  13 13  0,  12 14  0,  12 15  0,
```

```
                11 17  0,   9 18  0,  10 18  0,  -2 19  0,   9 19  0,  10 19  0,
                -2 20  0,   6 20  0,   9 20  0,  -2 21  0,   5 21  0,   5 24  0,
                -9-20  1,  -6-20  1,  -5-20  1, -10-19  1,  -9-19  1,  -6-19  1,
                 2-19  1, -11-18  1, -10-18  1,  -9-18  1, -11-17  1, -11-16  1,
                    (  248 More Missing: see the .ckf listing file)
PLAT971_ALERT_2_C Check Calcd Resid. Dens.  0.68Ang From Ag5A          2.28 eA-3
```

Author Response: This Alert is due to presence of residual density in the presence of heavy metal atom (Ag).

```
PLAT971_ALERT_2_C Check Calcd Resid. Dens.  0.25Ang From N15           2.21 eA-3
```

Author Response: This Alert is due to presence of residual density in the presence of heavy metal atom (Ag).

```
PLAT971_ALERT_2_C Check Calcd Resid. Dens.  1.01Ang From Ag2           2.18 eA-3
```

Author Response: This Alert is due to presence of residual density in the presence of heavy metal atom (Ag).

```
PLAT971_ALERT_2_C Check Calcd Resid. Dens.  0.21Ang From N16           2.15 eA-3
```

Author Response: This Alert is due to presence of residual density in the presence of heavy metal atom (Ag).

```
PLAT971_ALERT_2_C Check Calcd Resid. Dens.  0.46Ang From N36           2.01 eA-3
```

Author Response: This Alert is due to presence of residual density in the presence of heavy metal atom (Ag).

```
PLAT971_ALERT_2_C Check Calcd Resid. Dens.  0.71Ang From Ag6A          1.97 eA-3
```

Author Response: This Alert is due to presence of residual density in the presence of heavy metal atom (Ag).

```
PLAT971_ALERT_2_C Check Calcd Resid. Dens.  0.34Ang From N10           1.97 eA-3
```

Author Response: This Alert is due to presence of residual density in the presence of heavy metal atom (Ag).

```
PLAT971_ALERT_2_C Check Calcd Resid. Dens.  0.28Ang From C116          1.91 eA-3
```

Author Response: This Alert is due to presence of residual density in the presence of heavy metal atom (Ag).

PLAT971_ALERT_2_C Check Calcd Resid. Dens. 0.70Ang From F70A 1.85 eA-3

Author Response: This Alert is due to presence of residual density in the presence of heavy metal atom (Ag).

PLAT971_ALERT_2_C Check Calcd Resid. Dens. 0.91Ang From Ag3 1.84 eA-3

Author Response: This Alert is due to presence of residual density in the presence of heavy metal atom (Ag).

PLAT971_ALERT_2_C Check Calcd Resid. Dens. 1.04Ang From Ag1 1.84 eA-3

Author Response: This Alert is due to presence of residual density in the presence of heavy metal atom (Ag).

PLAT971_ALERT_2_C Check Calcd Resid. Dens. 1.09Ang From Ag2 1.81 eA-3

Author Response: This Alert is due to presence of residual density in the presence of heavy metal atom (Ag).

PLAT971_ALERT_2_C Check Calcd Resid. Dens. 0.98Ang From Ag3 1.81 eA-3

Author Response: This Alert is due to presence of residual density in the presence of heavy metal atom (Ag).

PLAT971_ALERT_2_C Check Calcd Resid. Dens. 1.08Ang From F36 1.75 eA-3

Author Response: This Alert is due to presence of residual density in the presence of heavy metal atom (Ag).

PLAT971_ALERT_2_C Check Calcd Resid. Dens. 0.67Ang From F69 1.71 eA-3

Author Response: This Alert is due to presence of residual density in the presence of heavy metal atom (Ag).

PLAT971_ALERT_2_C Check Calcd Resid. Dens. 0.64Ang From F51 1.70 eA-3

Author Response: This Alert is due to presence of residual density in the presence of heavy metal atom (Ag).

PLAT971_ALERT_2_C Check Calcd Resid. Dens. 0.75Ang From F71A 1.69 eA-3

Author Response: This Alert is due to presence of residual density in the presence of heavy metal atom (Ag).

```
PLAT971_ALERT_2_C Check Calcd Resid. Dens.  0.99Ang From Ag2            1.69 eA-3
```

Author Response: This Alert is due to presence of residual density in the presence of heavy metal atom (Ag).

```
PLAT971_ALERT_2_C Check Calcd Resid. Dens.  1.09Ang From Ag4            1.67 eA-3
```

Author Response: This Alert is due to presence of residual density in the presence of heavy metal atom (Ag).

```
PLAT971_ALERT_2_C Check Calcd Resid. Dens.  1.09Ang From Ag1            1.66 eA-3
```

Author Response: This Alert is due to presence of residual density in the presence of heavy metal atom (Ag).

```
PLAT971_ALERT_2_C Check Calcd Resid. Dens.  1.08Ang From F54            1.66 eA-3
```

Author Response: This Alert is due to presence of residual density in the presence of heavy metal atom (Ag).

```
PLAT971_ALERT_2_C Check Calcd Resid. Dens.  0.57Ang From N23A           1.66 eA-3
```

Author Response: This Alert is due to presence of residual density in the presence of heavy metal atom (Ag).

```
PLAT971_ALERT_2_C Check Calcd Resid. Dens.  1.00Ang From Ag12           1.64 eA-3
```

Author Response: This Alert is due to presence of residual density in the presence of heavy metal atom (Ag).

```
PLAT971_ALERT_2_C Check Calcd Resid. Dens.  1.36Ang From Ag1            1.63 eA-3
```

Author Response: This Alert is due to presence of residual density in the presence of heavy metal atom (Ag).

```
PLAT972_ALERT_2_C Check Calcd Resid. Dens.  0.16Ang From C49           -1.89 eA-3
PLAT972_ALERT_2_C Check Calcd Resid. Dens.  0.45Ang From Ag6A          -1.74 eA-3
PLAT972_ALERT_2_C Check Calcd Resid. Dens.  0.29Ang From F51           -1.74 eA-3
PLAT972_ALERT_2_C Check Calcd Resid. Dens.  1.47Ang From N6A           -1.73 eA-3
PLAT972_ALERT_2_C Check Calcd Resid. Dens.  0.51Ang From N23           -1.68 eA-3
PLAT972_ALERT_2_C Check Calcd Resid. Dens.  0.77Ang From Ag2           -1.68 eA-3
PLAT972_ALERT_2_C Check Calcd Resid. Dens.  0.27Ang From C15           -1.67 eA-3
```

```
PLAT972_ALERT_2_C Check Calcd Resid. Dens.  0.73Ang From Ag8          -1.62 eA-3
PLAT972_ALERT_2_C Check Calcd Resid. Dens.  0.29Ang From F71A         -1.60 eA-3
PLAT972_ALERT_2_C Check Calcd Resid. Dens.  0.81Ang From N28          -1.58 eA-3
PLAT972_ALERT_2_C Check Calcd Resid. Dens.  0.72Ang From Ag8          -1.57 eA-3
PLAT972_ALERT_2_C Check Calcd Resid. Dens.  1.52Ang From F6           -1.55 eA-3
PLAT972_ALERT_2_C Check Calcd Resid. Dens.  0.24Ang From F21          -1.55 eA-3
PLAT972_ALERT_2_C Check Calcd Resid. Dens.  0.31Ang From C50          -1.54 eA-3
PLAT972_ALERT_2_C Check Calcd Resid. Dens.  0.64Ang From Ag5          -1.53 eA-3
PLAT972_ALERT_2_C Check Calcd Resid. Dens.  0.50Ang From N13          -1.53 eA-3
PLAT977_ALERT_2_C Check Negative Difference Density on H4       .     -0.32 eA-3
PLAT977_ALERT_2_C Check Negative Difference Density on H24      .     -0.46 eA-3
PLAT977_ALERT_2_C Check Negative Difference Density on H25      .     -0.31 eA-3
PLAT977_ALERT_2_C Check Negative Difference Density on H76      .     -0.65 eA-3
PLAT977_ALERT_2_C Check Negative Difference Density on H89      .     -0.33 eA-3
PLAT977_ALERT_2_C Check Negative Difference Density on H149     .     -0.43 eA-3
PLAT977_ALERT_2_C Check Negative Difference Density on H150     .     -0.45 eA-3
PLAT977_ALERT_2_C Check Negative Difference Density on H18      .     -0.43 eA-3
PLAT977_ALERT_2_C Check Negative Difference Density on H33      .     -0.35 eA-3
PLAT977_ALERT_2_C Check Negative Difference Density on H113     .     -0.47 eA-3
PLAT977_ALERT_2_C Check Negative Difference Density on H124     .     -0.51 eA-3
```

Alert level G

```
FORMU01_ALERT_1_G  There is a discrepancy between the atom counts in the
            _chemical_formula_sum and _chemical_formula_moiety. This is
            usually due to the moiety formula being in the wrong format.
            Atom count from _chemical_formula_sum:   C108 H44 Ag12 Cl4 F72 N28 S4
            Atom count from _chemical_formula_moiety:C108.0019 H39.52959 Ag11.8335
PLAT002_ALERT_2_G Number of Distance or Angle Restraints on AtSite       266 Note
PLAT003_ALERT_2_G Number of Uiso or U(i,j)  Restrained non-H-Atoms       379 Report
PLAT007_ALERT_5_G Number of Unrefined Donor-H Atoms .............          8 Report
              H20   H26   H24   H32   H25B  H29A  H30A  H31
PLAT083_ALERT_2_G SHELXL Second Parameter in WGHT  Unusually Large    486.25 Why ?
PLAT154_ALERT_1_G The s.u.'s on the Cell Angles are Equal ..(Note)     0.001 Degree
PLAT171_ALERT_4_G The CIF-Embedded .res File Contains EADP Records         1 Report
PLAT172_ALERT_4_G The CIF-Embedded .res File Contains DFIX Records         1 Report
PLAT173_ALERT_4_G The CIF-Embedded .res File Contains DANG Records       132 Report
PLAT174_ALERT_4_G The CIF-Embedded .res File Contains FLAT Records        23 Report
PLAT176_ALERT_4_G The CIF-Embedded .res File Contains SADI Records        73 Report
PLAT177_ALERT_4_G The CIF-Embedded .res File Contains DELU Records        12 Report
PLAT178_ALERT_4_G The CIF-Embedded .res File Contains SIMU Records        12 Report
PLAT186_ALERT_4_G The CIF-Embedded .res File Contains ISOR Records        12 Report
PLAT188_ALERT_3_G A Non-default SIMU Restraint Value has been used    0.0100 Report
PLAT188_ALERT_3_G A Non-default SIMU Restraint Value has been used    0.0100 Report
PLAT188_ALERT_3_G A Non-default SIMU Restraint Value has been used    0.0100 Report
PLAT188_ALERT_3_G A Non-default SIMU Restraint Value has been used    0.0100 Report
PLAT188_ALERT_3_G A Non-default SIMU Restraint Value has been used    0.0100 Report
PLAT188_ALERT_3_G A Non-default SIMU Restraint Value has been used    0.0100 Report
PLAT188_ALERT_3_G A Non-default SIMU Restraint Value has been used    0.0100 Report
PLAT188_ALERT_3_G A Non-default SIMU Restraint Value has been used    0.0100 Report
PLAT188_ALERT_3_G A Non-default SIMU Restraint Value has been used    0.0100 Report
PLAT188_ALERT_3_G A Non-default SIMU Restraint Value has been used    0.0100 Report
PLAT188_ALERT_3_G A Non-default SIMU Restraint Value has been used    0.0100 Report
PLAT188_ALERT_3_G A Non-default SIMU Restraint Value has been used    0.0100 Report
PLAT191_ALERT_3_G A Non-default SADI Restraint Value has been used    0.0100 Report
PLAT191_ALERT_3_G A Non-default SADI Restraint Value has been used    0.0100 Report
PLAT191_ALERT_3_G A Non-default SADI Restraint Value has been used    0.0100 Report
```

```
PLAT191_ALERT_3_G A Non-default SADI Restraint Value has been used     0.0100 Report
PLAT191_ALERT_3_G A Non-default SADI Restraint Value has been used     0.0100 Report
PLAT191_ALERT_3_G A Non-default SADI Restraint Value has been used     0.0100 Report
PLAT191_ALERT_3_G A Non-default SADI Restraint Value has been used     0.0100 Report
PLAT191_ALERT_3_G A Non-default SADI Restraint Value has been used     0.0100 Report
PLAT191_ALERT_3_G A Non-default SADI Restraint Value has been used     0.0100 Report
PLAT191_ALERT_3_G A Non-default SADI Restraint Value has been used     0.0100 Report
PLAT191_ALERT_3_G A Non-default SADI Restraint Value has been used     0.0100 Report
PLAT191_ALERT_3_G A Non-default SADI Restraint Value has been used     0.0100 Report
PLAT191_ALERT_3_G A Non-default SADI Restraint Value has been used     0.0100 Report
PLAT191_ALERT_3_G A Non-default SADI Restraint Value has been used     0.0100 Report
PLAT191_ALERT_3_G A Non-default SADI Restraint Value has been used     0.0100 Report
PLAT191_ALERT_3_G A Non-default SADI Restraint Value has been used     0.0100 Report
PLAT191_ALERT_3_G A Non-default SADI Restraint Value has been used     0.0100 Report
PLAT191_ALERT_3_G A Non-default SADI Restraint Value has been used     0.0100 Report
PLAT191_ALERT_3_G A Non-default SADI Restraint Value has been used     0.0100 Report
PLAT191_ALERT_3_G A Non-default SADI Restraint Value has been used     0.0100 Report
PLAT191_ALERT_3_G A Non-default SADI Restraint Value has been used     0.0100 Report
PLAT191_ALERT_3_G A Non-default SADI Restraint Value has been used     0.0100 Report
PLAT191_ALERT_3_G A Non-default SADI Restraint Value has been used     0.0100 Report
PLAT191_ALERT_3_G A Non-default SADI Restraint Value has been used     0.0100 Report
PLAT191_ALERT_3_G A Non-default SADI Restraint Value has been used     0.0100 Report
PLAT191_ALERT_3_G A Non-default SADI Restraint Value has been used     0.0100 Report
PLAT191_ALERT_3_G A Non-default SADI Restraint Value has been used     0.0100 Report
PLAT191_ALERT_3_G A Non-default SADI Restraint Value has been used     0.0100 Report
PLAT191_ALERT_3_G A Non-default SADI Restraint Value has been used     0.0100 Report
PLAT191_ALERT_3_G A Non-default SADI Restraint Value has been used     0.0100 Report
PLAT191_ALERT_3_G A Non-default SADI Restraint Value has been used     0.0100 Report
PLAT191_ALERT_3_G A Non-default SADI Restraint Value has been used     0.0100 Report
PLAT191_ALERT_3_G A Non-default SADI Restraint Value has been used     0.0100 Report
PLAT191_ALERT_3_G A Non-default SADI Restraint Value has been used     0.0100 Report
PLAT191_ALERT_3_G A Non-default SADI Restraint Value has been used     0.0100 Report
PLAT191_ALERT_3_G A Non-default SADI Restraint Value has been used     0.0100 Report
PLAT191_ALERT_3_G A Non-default SADI Restraint Value has been used     0.0100 Report
PLAT191_ALERT_3_G A Non-default SADI Restraint Value has been used     0.0100 Report
PLAT191_ALERT_3_G A Non-default SADI Restraint Value has been used     0.0100 Report
PLAT191_ALERT_3_G A Non-default SADI Restraint Value has been used     0.0100 Report
PLAT191_ALERT_3_G A Non-default SADI Restraint Value has been used     0.0100 Report
PLAT191_ALERT_3_G A Non-default SADI Restraint Value has been used     0.0100 Report
PLAT191_ALERT_3_G A Non-default SADI Restraint Value has been used     0.0100 Report
PLAT191_ALERT_3_G A Non-default SADI Restraint Value has been used     0.0100 Report
PLAT191_ALERT_3_G A Non-default SADI Restraint Value has been used     0.0100 Report
PLAT191_ALERT_3_G A Non-default SADI Restraint Value has been used     0.0100 Report
PLAT191_ALERT_3_G A Non-default SADI Restraint Value has been used     0.0100 Report
PLAT191_ALERT_3_G A Non-default SADI Restraint Value has been used     0.0100 Report
PLAT191_ALERT_3_G A Non-default SADI Restraint Value has been used     0.0100 Report
PLAT191_ALERT_3_G A Non-default SADI Restraint Value has been used     0.0100 Report
PLAT191_ALERT_3_G A Non-default SADI Restraint Value has been used     0.0100 Report
PLAT191_ALERT_3_G A Non-default SADI Restraint Value has been used     0.0100 Report
PLAT191_ALERT_3_G A Non-default SADI Restraint Value has been used     0.0100 Report
PLAT191_ALERT_3_G A Non-default SADI Restraint Value has been used     0.0100 Report
PLAT191_ALERT_3_G A Non-default SADI Restraint Value has been used     0.0100 Report
PLAT191_ALERT_3_G A Non-default SADI Restraint Value has been used     0.0100 Report
PLAT191_ALERT_3_G A Non-default SADI Restraint Value has been used     0.0100 Report
PLAT191_ALERT_3_G A Non-default SADI Restraint Value has been used     0.0100 Report
PLAT191_ALERT_3_G A Non-default SADI Restraint Value has been used     0.0100 Report
PLAT191_ALERT_3_G A Non-default SADI Restraint Value has been used     0.0100 Report
```

```
PLAT191_ALERT_3_G A Non-default SADI Restraint Value has been used      0.0100 Report
PLAT191_ALERT_3_G A Non-default SADI Restraint Value has been used      0.0100 Report
PLAT191_ALERT_3_G A Non-default SADI Restraint Value has been used      0.0100 Report
PLAT191_ALERT_3_G A Non-default SADI Restraint Value has been used      0.0100 Report
PLAT191_ALERT_3_G A Non-default SADI Restraint Value has been used      0.0100 Report
PLAT191_ALERT_3_G A Non-default SADI Restraint Value has been used      0.0100 Report
PLAT191_ALERT_3_G A Non-default SADI Restraint Value has been used      0.0100 Report
PLAT191_ALERT_3_G A Non-default SADI Restraint Value has been used      0.0100 Report
PLAT191_ALERT_3_G A Non-default SADI Restraint Value has been used      0.0100 Report
PLAT191_ALERT_3_G A Non-default SADI Restraint Value has been used      0.0100 Report
PLAT191_ALERT_3_G A Non-default SADI Restraint Value has been used      0.0100 Report
PLAT191_ALERT_3_G A Non-default SADI Restraint Value has been used      0.0100 Report
PLAT191_ALERT_3_G A Non-default SADI Restraint Value has been used      0.0100 Report
PLAT192_ALERT_3_G A Non-default DELU Restraint Value for SecondPar      0.0200 Report
PLAT192_ALERT_3_G A Non-default DELU Restraint Value for SecondPar      0.0200 Report
PLAT192_ALERT_3_G A Non-default DELU Restraint Value for SecondPar      0.0200 Report
PLAT192_ALERT_3_G A Non-default DELU Restraint Value for SecondPar      0.0200 Report
PLAT192_ALERT_3_G A Non-default DELU Restraint Value for SecondPar      0.0200 Report
PLAT192_ALERT_3_G A Non-default DELU Restraint Value for SecondPar      0.0200 Report
PLAT192_ALERT_3_G A Non-default DELU Restraint Value for SecondPar      0.0200 Report
PLAT192_ALERT_3_G A Non-default DELU Restraint Value for SecondPar      0.0200 Report
PLAT192_ALERT_3_G A Non-default DELU Restraint Value for SecondPar      0.0200 Report
PLAT192_ALERT_3_G A Non-default DELU Restraint Value for SecondPar      0.0200 Report
PLAT192_ALERT_3_G A Non-default DELU Restraint Value for SecondPar      0.0200 Report
PLAT192_ALERT_3_G A Non-default DELU Restraint Value for SecondPar      0.0200 Report
PLAT242_ALERT_2_G Low    'MainMol' Ueq as Compared to Neighbors of         C46 Check
PLAT242_ALERT_2_G Low    'MainMol' Ueq as Compared to Neighbors of         C59 Check
PLAT242_ALERT_2_G Low    'MainMol' Ueq as Compared to Neighbors of         C75 Check
PLAT242_ALERT_2_G Low    'MainMol' Ueq as Compared to Neighbors of        C105 Check
PLAT242_ALERT_2_G Low    'MainMol' Ueq as Compared to Neighbors of         C38 Check
PLAT242_ALERT_2_G Low    'MainMol' Ueq as Compared to Neighbors of         C39 Check
PLAT242_ALERT_2_G Low    'MainMol' Ueq as Compared to Neighbors of         C70 Check
PLAT242_ALERT_2_G Low    'MainMol' Ueq as Compared to Neighbors of         C96 Check
PLAT242_ALERT_2_G Low    'MainMol' Ueq as Compared to Neighbors of         C97 Check
PLAT242_ALERT_2_G Low    'MainMol' Ueq as Compared to Neighbors of        C101 Check
PLAT301_ALERT_3_G Main Residue  Disorder .............(Resd    1)        100% Note
PLAT302_ALERT_4_G Anion/Solvent/Minor-Residue Disorder (Resd    2)         10% Note
PLAT302_ALERT_4_G Anion/Solvent/Minor-Residue Disorder (Resd    3)          7% Note
PLAT302_ALERT_4_G Anion/Solvent/Minor-Residue Disorder (Resd    4)        100% Note
PLAT302_ALERT_4_G Anion/Solvent/Minor-Residue Disorder (Resd    5)        100% Note
PLAT302_ALERT_4_G Anion/Solvent/Minor-Residue Disorder (Resd    6)        100% Note
PLAT302_ALERT_4_G Anion/Solvent/Minor-Residue Disorder (Resd    7)        100% Note
PLAT302_ALERT_4_G Anion/Solvent/Minor-Residue Disorder (Resd    8)        100% Note
PLAT302_ALERT_4_G Anion/Solvent/Minor-Residue Disorder (Resd    9)        100% Note
PLAT302_ALERT_4_G Anion/Solvent/Minor-Residue Disorder (Resd   10)        100% Note
PLAT302_ALERT_4_G Anion/Solvent/Minor-Residue Disorder (Resd   11)        100% Note
PLAT302_ALERT_4_G Anion/Solvent/Minor-Residue Disorder (Resd   12)        100% Note
PLAT302_ALERT_4_G Anion/Solvent/Minor-Residue Disorder (Resd   13)        100% Note
PLAT304_ALERT_4_G Non-Integer Number of Atoms in ..... (Resd    1)       55.60 Check
PLAT304_ALERT_4_G Non-Integer Number of Atoms in ..... (Resd    4)       38.34 Check
PLAT304_ALERT_4_G Non-Integer Number of Atoms in ..... (Resd    5)        2.48 Check
PLAT304_ALERT_4_G Non-Integer Number of Atoms in ..... (Resd    6)        6.66 Check
PLAT304_ALERT_4_G Non-Integer Number of Atoms in ..... (Resd    7)       12.81 Check
PLAT304_ALERT_4_G Non-Integer Number of Atoms in ..... (Resd    8)       15.25 Check
PLAT304_ALERT_4_G Non-Integer Number of Atoms in ..... (Resd    9)       15.25 Check
PLAT304_ALERT_4_G Non-Integer Number of Atoms in ..... (Resd   10)        7.75 Check
PLAT304_ALERT_4_G Non-Integer Number of Atoms in ..... (Resd   11)       10.19 Check
```

```
PLAT304_ALERT_4_G Non-Integer Number of Atoms in ..... (Resd   12)       7.75 Check
PLAT304_ALERT_4_G Non-Integer Number of Atoms in ..... (Resd   13)       9.91 Check
PLAT432_ALERT_2_G Short Inter X...Y Contact  F23A     ..C9        .       2.55 Ang.
                                                -x,2-y,2-z  =     2_577 Check
PLAT432_ALERT_2_G Short Inter X...Y Contact  F23A     ..C101      .       2.80 Ang.
                                                     x,y,z  =     1_555 Check
PLAT432_ALERT_2_G Short Inter X...Y Contact  F82      ..C46       .       2.63 Ang.
                                                -x,1-y,1-z  =     2_566 Check
PLAT432_ALERT_2_G Short Inter X...Y Contact  F82      ..C15       .       2.67 Ang.
                                                -x,1-y,1-z  =     2_566 Check
PLAT432_ALERT_2_G Short Inter X...Y Contact  F82      ..C10       .       2.79 Ang.
                                                -x,1-y,1-z  =     2_566 Check
PLAT432_ALERT_2_G Short Inter X...Y Contact  N9       ..C47       .       2.92 Ang.
                                                   x,y,1+z  =     1_556 Check
PLAT432_ALERT_2_G Short Inter X...Y Contact  N19      ..C47       .       2.99 Ang.
                                                   x,y,1+z  =     1_556 Check
PLAT434_ALERT_2_G Short Inter HL..HL Contact F12      ..F71       .       2.78 Ang.
                                                  -1+x,y,z  =     1_455 Check
PLAT434_ALERT_2_G Short Inter HL..HL Contact F20      ..F68       .       2.75 Ang.
                                                   1+x,y,z  =     1_655 Check
PLAT434_ALERT_2_G Short Inter HL..HL Contact F24      ..F63A      .       2.68 Ang.
                                               1-x,1-y,2-z  =     2_667 Check
PLAT434_ALERT_2_G Short Inter HL..HL Contact F27      ..F69A      .       2.82 Ang.
                                                -x,2-y,2-z  =     2_577 Check
PLAT434_ALERT_2_G Short Inter HL..HL Contact F41      ..F82       .       1.76 Ang.
                                                -x,1-y,1-z  =     2_566 Check
PLAT434_ALERT_2_G Short Inter HL..HL Contact F66      ..F21A      .       2.78 Ang.
                                                     x,y,z  =     1_555 Check
PLAT790_ALERT_4_G Centre of Gravity not Within Unit-Cell: Resd.  #          3 Note
              C15 H3 Ag3 F18 N6
PLAT790_ALERT_4_G Centre of Gravity not Within Unit-Cell: Resd.  #          9 Note
              C12 H8 Cl N S
PLAT790_ALERT_4_G Centre of Gravity not Within Unit-Cell: Resd.  #         10 Note
              C12 H8 Cl N S
PLAT811_ALERT_5_G No ADDSYM Analysis: Too Many Excluded Atoms ....          ! Info
PLAT860_ALERT_3_G Number of Least-Squares Restraints ............       9142 Note
PLAT910_ALERT_3_G Missing FCF Reflection(s) Below Theta(Min)[Deg]=       2.29 Note
                0  1  0,   0  0  1,
PLAT912_ALERT_4_G Missing # of FCF Reflections Above STh/L=  0.600       1408 Note
PLAT913_ALERT_3_G Missing # of Very Strong Reflections in FCF ....          2 Note
                0  0  4,   0 -2  8,
PLAT933_ALERT_2_G Number of HKL-OMIT Records in Embedded .res File         99 Note
              -14-12  1, -13-14  1, -12-15  1, -12-15  2, -11-18 17, -11-16  1,
              -11-16  2,  -8-21 10,  -8-19  2,  -6-19  1,  -4-23  8,  -3-25  4,
               -3-24  5,  -3-23  9,  -3-22 10,  -2-25  7,  -2-25  8,  -2-24  8,
               -2-23  8,  -2-23  9,  -2-23 10,  -2-22 10,  -2-22 11,  -2-21 11,
               -2-19 12,  -1-25  6,  -1-24  8,  -1-21 12,  -1-20 13,   0-24  9,
                0-21 12,   0-20 12,   1-24  7,   1-23  8,   3-25  9,   4 25  1,
                4 25  2,   5 24  0,   5 24  1,   6 19  2,   6 19  4,   6 23  1,
                6 23  2,   6 23  4,   7  4  3,   7 17 10,   8 11 17,   8 15  2,
                8 15 12,   8 19  3,
PLAT941_ALERT_3_G Average HKL Measurement Multiplicity ..........        2.3 Low
PLAT969_ALERT_5_G The 'Henn et al.' R-Factor-gap value ..........      4.429 Note
              Predicted wR2: Based on SigI**2  6.03 or SHELX Weight 25.49
PLAT978_ALERT_2_G Number C-C Bonds with Positive Residual Density.          1 Info
```

  0 **ALERT level A** = Most likely a serious problem - resolve or explain
  2 **ALERT level B** = A potentially serious problem, consider carefully
 66 **ALERT level C** = Check. Ensure it is not caused by an omission or oversight
170 **ALERT level G** = General information/check it is not something unexpected

  6 ALERT type 1 CIF construction/syntax error, inconsistent or missing data
 85 ALERT type 2 Indicator that the structure model may be wrong or deficient
109 ALERT type 3 Indicator that the structure quality may be low
 35 ALERT type 4 Improvement, methodology, query or suggestion
  3 ALERT type 5 Informative message, check

Validation response form

Please find below a validation response form (VRF) that can be filled in and pasted into your CIF.

```
# start Validation Reply Form
_vrf_PLAT042_lcz088
;
PROBLEM: Calc. and Reported MoietyFormula Strings  Differ     Please Check
RESPONSE: ...
;
_vrf_PLAT082_lcz088
;
PROBLEM: High R1 Value ..................................       0.11 Report
RESPONSE: ...
;
_vrf_PLAT084_lcz088
;
PROBLEM: High wR2 Value (i.e. > 0.25) ...................       0.27 Report
RESPONSE: ...
;
_vrf_PLAT088_lcz088
;
PROBLEM: Poor Data / Parameter Ratio ....................       9.27 Note
RESPONSE: ...
;
_vrf_PLAT250_lcz088
;
PROBLEM: Large U3/U1 Ratio for <U(i,j)> Tensor(Resd    2)        2.8 Note
RESPONSE: ...
;
_vrf_PLAT362_lcz088
;
PROBLEM: Short  C(sp3)-C(sp2) Bond  C10      - C46      .       1.41 Ang.
RESPONSE: ...
;
_vrf_PLAT721_lcz088
;
PROBLEM: Bond    Calc     1.37(7), Rep     1.39000 Dev...       0.02 Ang.
RESPONSE: ...
;
_vrf_PLAT723_lcz088
;
PROBLEM: Torsion Calc      -2(10), Rep        0.00 Dev...       2.00 Sigma
RESPONSE: ...
```

```
;
_vrf_PLAT906_lcz088
;
PROBLEM: Large K Value in the Analysis of Variance ......     28.898 Check
RESPONSE: ...
;
_vrf_PLAT911_lcz088
;
PROBLEM: Missing FCF Refl Between Thmin & STh/L=    0.600        278 Report
RESPONSE: ...
;
_vrf_PLAT972_lcz088
;
PROBLEM: Check Calcd Resid. Dens.  0.16Ang From C49           -1.89 eA-3
RESPONSE: ...
;
_vrf_PLAT977_lcz088
;
PROBLEM: Check Negative Difference Density on H4        .      -0.32 eA-3
RESPONSE: ...
;
# end Validation Reply Form
```

It is advisable to attempt to resolve as many as possible of the alerts in all categories. Often the minor alerts point to easily fixed oversights, errors and omissions in your CIF or refinement strategy, so attention to these fine details can be worthwhile. It is up to the individual to critically assess their own results and, if necessary, seek expert advice.

PLATON version of 17/09/2025; check.def file version of 17/09/2025

---

duplicate check

No duplication found

Datablock lcz088 - ellipsoid plot

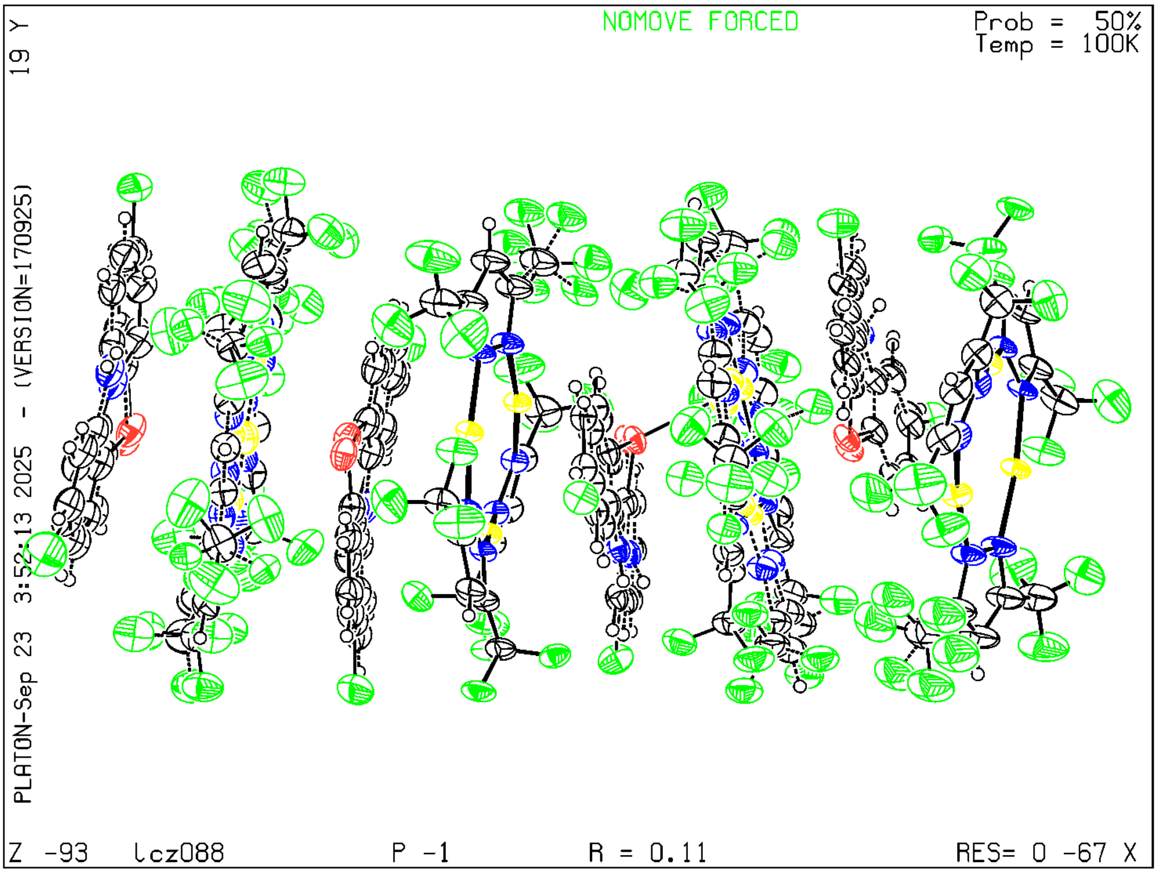

Supplement: Supplementary file 2 — Supporting File 2: anie72462‐sup‐0002‐DataFile.zip. [file ANIE-65-e1218503-s002.zip › supplementary_materials_crystallographic_data submit.zip!/cif_and_checkcif(1-114)/Ag3Pz3_88/Ag3Pz3_88_checkcif.pdf]
